# Improvement in Tensile Quasi-Static and Fatigue Properties of Carbon Fiber-Reinforced Epoxy Laminates with Matrices Modified by Carbon Nanotubes and Graphene Nanoplatelets Hybrid Nanofillers

**DOI:** 10.3390/nano11123459

**Published:** 2021-12-20

**Authors:** Yi-Ming Jen, Yu-Ching Huang

**Affiliations:** Department of Mechanical and Mechatronic Engineering, National Taiwan Ocean University, Keelung City 202301, Taiwan; 10772011@email.ntou.edu.tw

**Keywords:** carbon fiber-reinforced epoxy (CFEP) laminate, multiwalled carbon nanotube (MWCNT), graphene nanoplatelets (GNP), static strength, fatigue strength, fracture toughness, synergistic effect, bridging effect, crack deflection effect

## Abstract

The monotonic and cyclic properties of carbon fiber-reinforced epoxy (CFEP) laminate specimens with matrices modified by multiwalled carbon nanotubes (MWCNTs) and graphene nanoplatelets (GNPs) were experimentally studied. The laminate specimens were fabricated by the hand lay-up procedure and six MWCNT:GNP weight ratios, i.e., 0:0, 10:0, 0:10, 5:5, 9:1, and 1:9, were considered to prepare the nanoparticle-modified epoxy resin by using an ultrasonic homogenizer and a planetary centrifugal mixer. Then, these laminate specimens with their matrices modified under various nanofiller ratios were employed to investigate the influence of the number of nanofiller types and hybrid nanofiller ratios on the quasi-static strength, fatigue strength, and mode I fracture toughness. The experimental results show that adding individual types of nanoparticles has a slight influence on the quasi-static and fatigue strengths of the CFEP laminates. However, the remarkable synergistic effect of MWCNTs and GNPs on the studied mechanical properties of the CFEP laminates with matrices reinforced by hybrid nanoparticles has been observed. Examining the evolution of stiffness-based degradation indicates that adding hybrid nanoparticles to the matrix can reduce the degradation effectively. The high experimental data of the mode I fracture toughness of hybrid nano-CFEP laminates demonstrate that embedding hybrid nanoparticles in the matrix is beneficial to the interlaminar properties, further improving the fatigue strength. The pushout mechanism of the MWCNTs and the crack deflection effect of the GNPs suppress the growth and linkage of microcracks in the matrix. Furthermore, the bridging effect of the nanoparticles at the fiber/matrix interface retards the interfacial debonding, further improving the resistance to delamination propagation.

## 1. Introduction

Due to the characteristics of high specific stiffness and strength, carbon fiber-reinforced polymer (CFRP) laminates have been widely applied in the structures of vehicles, aircrafts, ships, and wind turbines. As a novel engineering material, the CFRP composites are frequently subjected to the fluctuating loads and, therefore, the knowledge of the fatigue design of the CFRP laminates is demanded urgently. In general, the fatigue failure process of FRP composites includes five major stages: matrix cracking, interfacial debonding, delamination, fiber fracture, and final failure [1,2]. Many efforts have been made to improve the fatigue strength of FRP laminates by interrupting or retarding the evolution of the failure mechanism [3,4]. Moreover, due to the characteristics of the laminates, FRP composites have high in-plane strength, but the out-of-plane properties are not as expected. Accordingly, many strategies, such as stitching [5,6], z-pinning [7,8,9], three-dimensional (3D) weaving [10,11], and matrix toughing [12,13], have been applied recently to improve the interlaminar strength of FRPs. Among these techniques regarding enhancing the out-of-plane properties of laminates, matrix toughing has received much attention, for the other ones remarkably decrease the in-plane strengths. With the advancement of manufacturing technology and synthesizing nano-sized materials, the technique of adding nanoparticles in the matrix has been employed extensively to improve the quasi-static strength, fracture toughness, interlaminar shear strength (ILSS), and post-impact strength of the FRP composites [14,15,16,17,18,19,20,21]. Surveying past studies regarding the mechanical properties of the nano-modified FRP composites, the fatigue resistance of the nanoparticle-modified FRP composites has been rarely investigated. In practical applications, FRP composites are frequently subjected to cyclic loading. Accordingly, the application of nanoparticles in the improvement of the fatigue resistance of FRPs has, recently, gradually received attention. Dispersing nanoparticles in the matrix has been attempted to prevent the matrix cracking formed in the early stage, and the subsequent delamination, further improving the fatigue strength.

Carbon nanotubes (CNTs) are the most frequently applied carbon nanoparticle to reinforce the polymer matrix. The characteristics of their one-dimensional (1D) structures make CNTs excellent reinforcements to improve mechanical properties by a bridging effect. In 2008, Grimmer and Dharan [22] studied the fatigue strength of glass fiber-reinforced polymer (GFRP) laminates modified by 1% multiwalled CNTs (MWCNTs). Compared to the neat GFRP specimens, the MWCNT-modified GFRP laminates present significant improvement in fatigue strength. The effect of fluorine-functionalized CNTs on the fatigue strength of carbon fiber-reinforced epoxy (CFEP) laminates was studied by Davis et al. in 2010 [23]. The nanoparticles were sprayed on both sides of the fabrics to prepare the specimens. The tension–tension fatigue strength of the nanoparticle-modified CFRP specimens increased with concentrations of CNTs up to 0.5 wt.%. However, no improvement in fatigue strength was observed when the CNT-modified CFRP specimens were fatigue tested under fully reversed cyclic loading. In the same year, Chang [24] examined the flexural fatigue strength of MWCNT-modified carbon/epoxy and glass/epoxy laminate composites. The CFRP and GFRP specimens, modified with various contents of MWCNTs, were fatigue tested under the same stress range. Experimental results show that the addition of MWCNTs increases the fatigue lives of CFRP and GFRP laminates significantly. Moreover, the fatigue life of MWCNT-modified FRPs increases with the contents of MWCNTs, up to 2.0 phr. In the same year, the effect of the stress ratio on the fatigue strength of the glass/epoxy laminates modified by 0.3 wt.% MWCNTs was investigated by Böger et al. [25]. Takeda et al. [26] explored the cryogenic fatigue resistance of the glass/epoxy laminates modified by 0.5 wt.% MWCNTs and 10 phr n-butyl glycidyl ether (n-BGE) at 77 K. Experimental results show that adding MWCNTs or n-BGE individually reduces the fatigue life of studied GFRP laminates when the specimens were fatigue tested under the maximum stress of 500 MPa. However, the synergistic effect of MWCNTs and n-BGE was observed in the improvement of the fatigue strength. In 2014, Borrogo et al. [27] examined the effect of added CNTs on the fatigue strength of glass fiber-reinforced epoxy laminates. Experimental results demonstrate that the GFRP specimens with 0.5 wt.% CNTs present slightly higher fatigue resistances than the neat GFRP composites. However, adding 1 wt.% CNTs to the matrix is detrimental to the fatigue strength of the studied GFRP laminates, due to the agglomeration of nanoparticles.

The graphene-based nanofillers are other commonly applied reinforcing particles. Their two-dimensional (2D) flake-like shape provides a large contact area with the polymer matrix to enhance the crosslink between the graphene and the polymer. Moreover, the crack deflects the direction of propagation when it encounters these graphene-based nanoparticles. The additional energy consumption due to the crack deflection effect reduces the fatigue crack growth rate. In 2010, Yavari et al. [28] studied the effect of hierarchical graphene platelets (GPLs) on the tensile and bending-fatigue strengths of glass/epoxy composite specimens. The studied GFRP specimens with various contents of GPLs (0.05–0.2 wt.%) were prepared and bending-fatigue tested. Experimental results display that the flexural fatigue strength of GPL-modified GFRP laminates increases with the concentrations of the GPLs. Furthermore, controlling the concentration of nanoparticles at 0.2 wt.%, the flexural fatigue strength of the GFRP specimens modified by GPLs is higher than those modified by single-walled CNTs (SWCNTs) or MWCNTs. Furthermore, the modification with 0.2 wt.% GPLs is also beneficial to the tensile fatigue strength of glass/epoxy laminates. Experimental results reveal that in all fatigue tests with various stress ratios (0.1, −1, or 10), adding 0.3 wt.% MWCNTs can increase the fatigue strength of the GFRP specimens effectively. The fatigue lives at the same stress levels were increased by several orders. In 2013, Shen et al. [29] made a comparison of fatigue strengths between the neat carbon/epoxy laminates and the CFRP specimens with matrices experimentally modified by 0.25 wt.% graphene nanoplatelets (GNPs). The experimental results show that adding GNPs to the matrix can improve the fatigue strength of CFRP significantly. Compared to the GNP-modified CFRP laminates, the deeper slope of the S–N curve for the neat composites indicates that the fatigue strength of neat CFRP laminates is more sensitive to the applied stress levels. In the next year, Knoll et al. [4] examined the effect of adding 0.3 wt.% few-layered graphene (FLG) and MWCNTs to the matrix on the fatigue strength of carbon/epoxy laminates. Dispersion of nanoparticles was found to increase the fatigue strength of CFRP composites. Moreover, the laminates modified by FLG had higher fatigue resistances than those modified by MWCNTs. The addition of nanoparticles was found to shift the degradation curves of CFRPs successfully by suspending the development of damage and further enhancing the fatigue strength. In 2018, the S–N curves of antisymmetric glass/epoxy laminates (+45/0_2_/90_2_/0_2_/−45°), modified by 0.1 wt.% SWCNTs and 0.1 wt.% GNPs, were established experimentally by Bourchak et al. [30]. Comparing this with the fatigue strength of neat antisymmetric GFRP specimens displays that the modification of nanoparticles markedly increases the fatigue strength of the GFRP composites. Furthermore, the SWCNTs presented a more pronounced improvement in fatigue strength than SWCNTs. Leopold et al. [31] studied the effect of an addition of 0.3 wt.% FLG on the fatigue strength of the (0, 90°)_2s_ cross-ply carbon/epoxy laminates in 2019. The experimental results of the fully-reversed fatigue tests show that the nanoparticle modification at the 0° layers decreases the fatigue strength slightly. However, the laminates with nanoparticles modified at the 90° layers present much lower fatigue strengths than the neat CFRP specimens. In 2020, the fatigue strength of carbon/epoxy laminates reinforced with 0.1 wt.% GNPs was experimentally investigated by Tareq et al. [32]. Dispersing few amounts of GNPs in the matrix increases the mean fatigue life of the studied CFRP laminate specimens by 155%.

Except for the fatigue behavior of bulk FRP specimens, the effect of nanoparticle modification on the fatigue crack propagation rate of FRP was rarely studied. In 2010, Grimmer and Dharan [33] found that adding 1 wt.% MWCNTs to the matrix can significantly reduce the fatigue delamination growth rate of glass fiber-reinforced epoxy laminates. Romhány and Szebényi [34] studied the mode I fatigue crack growth rate of the carbon/epoxy composites containing 0.3 wt.% MWCNTs in the same year. Experimental results show that the reinforcement of MWCNTs decreases the crack growth rate of the CFRP laminates by 69%. In 2014, Fenner and Daniel [35] examined the mode I fatigue delamination propagation rate of woven carbon fiber-reinforced epoxy laminate composites with matrices modified by 0.5 wt.% MWCNTs. Dispersing MWCNTs in the matrix was found to reduce the delamination growth rate by factor of 2. Kadlec et al. [36] investigated the mode I fatigue delamination growth rate of the carbon/epoxy laminates with matrices reinforced by 0.5 wt.% MWCNTs in 2016. Mixing MWCNTs in the epoxy resin can decrease the delamination propagation rate of the studied CFRP specimens by 80%.

Surveying the past investigations, regarding the fatigue strength of nanoparticle-modified FRPs, indicates that most studies adopted individual types of carbon nanoparticles to enhance the fatigue resistance of FRP. Although the synergistic effect of hybrid nanoparticles on the mechanical properties of polymer nanocomposites has been explored in the past ten years [37,38,39,40,41], the fatigue characteristics of the FRP specimens containing hybrid nanoparticles have not been studied until recently. The benefits of combining the dissimilar characteristics of nanoparticles with different dimensionalities to improve the fatigue strength of FRPs are worthy of being studied. Hence, the tensile quasi-static and fatigue strengths of the unidirectional CFEP laminates containing MWCNTs and GNPs were experimentally investigated herein to explore the synergistic effect of these hybrid nanoparticles on the mechanical properties of the bulk composite specimens. Moreover, the resistance to the delamination propagation is dependent on the interlaminar property of the FRP composites. The mode I interlaminar fracture toughness tests were also performed on the studied nano-modified specimens to examine the influence of the hybrid nanoparticle system on the interlaminar strength. The weight ratio between the two employed nanoparticles was considered as a main variable of the study. The fracture surfaces of the specimens were also observed to identify the enhancement mechanism of the nanoparticles on the fatigue performance.

## 2. Materials and Methods

### 2.1. Materials

**Figure 1:**
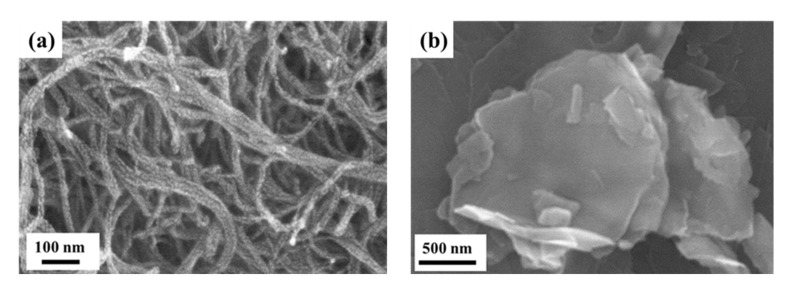
SEM images of the as-received (**a**) MWCNTs and (**b**) GNPs.

The unidirectional 12 k carbon fabrics used in this study were provided by Formosa Taffeta Co. Ltd., Touliou, Taiwan, with the designation of ECCFM. The areal weight and thickness of the fabrics were 216 g/m^2^ and 0.28 mm, respectively. The solvent-type epoxy, designated EPO-622 (Epotech Composite Co., Taichung, Taiwan), was utilized to prepare the matrix material. The epoxy solvent was composed of epoxy resin, a dicyanamide curing agent, and a methyl ethyl ketone (MEK) solvent. The ratio between the epoxy resin and curing agent in weight was 18:2. The MWCNT used in this study was fabricated by Nanocyl, Sambreville, Belgium, with the designation of NC7000. The SEM image of the employed MWCNTs is shown in Figure 1a. The diameter and length of the as-received MWCNTs were approximately 9.5 nm and 1.5 μm, respectively. The purity was higher than 90%. The GNPs employed in the study were supplied by Knano Co., Xiamen, China, with the designation of KNG-150. Figure 1b displays the SEM image of the utilized GNPs. The thickness ranged from 5 to 15 nm, and the diameter was about 5 μm. The purity was higher than 99.5%. These as-received nanoparticles, MWCNT and GNPs, were first modified using Maleic anhydride to establish beneficial functional groups on the surfaces of the nanoparticles.

### 2.2. Nano-Modified Epoxies

**Figure 2:**
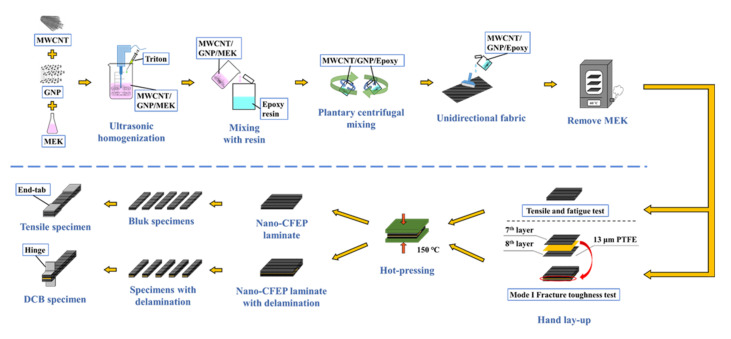
Procedure of specimen preparation.

The procedure of specimen preparation is schematically illustrated in Figure 2. To prepare the nano-modified epoxy resin, the nanoparticles with the required amounts were first dried at 120 °C for 1 h to remove the moisture. The dried nanoparticles were then mixed with the MEK solvent and stirred for 10 min using an ultrasonic homogenizer. The ultrasonic homogenizer provided more energy and made a more uniform dispersion of the nanoparticles in the MEK than the ultrasonic bath. Instead of the energy being spread diffusely, the nanoparticles surrounding the probe were blasted with an enormous amount of energy. Accordingly, the nanoparticles were dispersed rapidly. Next the suspension was mixed with the surfactant Triton X-405 (Sigma–Aldrich, Inc., St. Louis, MO, USA) and sonicated continuously for another 20 min. The employed non-ionic surfactants can increase the stabilization of the carbon nanoparticles in the solvent through their absorption, further improving the dispersion of the nanoparticles. Then, the mixture was subsequently added into the solvent-type epoxy and agitated using a planetary centrifugal mixer for 20 min to obtain a uniform dispersion of nanoparticles in the blend. Due to the high-power rotating centrifugal force, a more uniform dispersion of the nanoparticles in the blend can be obtained by using a planetary centrifugal mixer instead of traditional mechanical stirring techniques. The total weight percentage of two nanoparticles in the epoxy resin was controlled at 0.25 wt.%.

### 2.3. Fabrication of Nano-CFEP Laminate Specimens

The CFEP laminates were fabricated using the hand lay-up procedure with the fiber volume fraction controlled at 45%. The aforementioned nanoparticle/epoxy mixture was poured onto the fabric and squeegeed uniformly using a roller and a scraper. Then, the fabric was dried at 60 °C to remove the MEK solvent and obtain the impregnated fabric. To prepare two types of specimens, two prepreg lay-ups were considered to prepare the specimens. For the specimens employed in the quasi-static and fatigue tests, four plies of impregnated fabrics were laid-up with the fiber direction of (0)_4_. The other type of lay-up was for the specimens used in the mode I fracture toughness tests. One strip of polytetrafluoroethylene (PTFE) release film with a thickness of 13 μm was inserted between the 7th and 8th ply of the fabrics to create an artificial delamination in the (0)_14_ laminates. Next, the prepregs were hot-pressed at 150 °C in a vacuum to obtain the laminate plates. The pressure was increased gradually to 1300 psi and remained unchanged for 20 min. The cured laminate plates were then cooled down at room temperature. The laminate plates with different lay-ups were cut according to the ASTM (American Society for Testing and Materials) standards D3039 [42] and D5528 [43], respectively, to obtain the bulk specimens for the quasi-static/fatigue tests and the double cantilever beam (DCB) specimens for the mode I fracture toughness tests. Six hybrid nanofiller ratios, i.e., MWCNT:GNP = 0:0, 10:0, 0:10, 9:1, 5:5, and 1:9 were considered in the matrix modification of the bulk and DCB specimens to study the effect of the nanofiller ratio on the studied mechanical properties of the CFEP laminate specimens. The specimens with a matrix modified under the MWCNT:GNP ratios of 10:0 and 0:10 represent the ones with a matrix containing only MWCNTs and only GNPs, respectively. The CFEP laminate specimens with a nanofiller ratio of 0:0 are the referential ones, with neat epoxy matrices.

**Figure 3:**
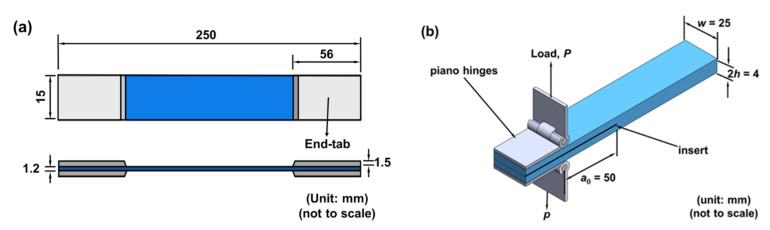
Shape and dimensions of the (**a**) bulk specimens for the static and fatigue tests, and (**b**) DCB specimens for the mode I fracture toughness tests.

For the specimens used in the quasi-static and fatigue tests, four aluminum end-tabs with a thickness of 1.5 mm were bonded on the specimens for clamping by the grips. The shape and dimensions of the tension specimen are shown in Figure 3a. Similarly, two piano hinges were bonded onto the DCB specimens for clamping by the grips in the fracture toughness tests, as shown in Figure 3b. The sides of the pre-delaminated DCB specimens were painted with white correction fluid, and the scales were marked for observation of the delamination length.

### 2.4. Tests of Mechanical Properties

All tests of the mechanical properties of the studied specimens were performed using a servo-hydraulic MTS 810 material testing system (MTS Systems Corporation; Eden Prairie, MN, USA). First, the quasi-static tests were conducted, according to the ASTM standard D3039 [33], to obtain the static mechanical properties of the studied nano-CFEP composites modified under various nanofiller ratios. The specimen was monotonically pulled to separation at room temperature, under displacement controlled with a crosshead speed of 2 mm/min. The engineering stress *σ* was obtained as the applied load divided by the cross-sectional area of the gage length region, and the strain *ε* was measured using an extensometer with a gage length of 20 mm (632.31F-24, MTS Systems Corporation; Eden Prairie, MN, USA). Three to five identical tests were performed repeatedly for each type of specimen to make sure the obtained experimental data were reliable.

The fatigue tests were performed according to the standard of ASTM D3479 [44]. The tests were carried out in the load-controlled mode. The waveform of the applied cyclic load was sinusoidal, with a frequency of 10 Hz. The stress ratio, *R*, defined as the minimum stress *σ*_min_ divided by the maximum stress *σ*_max_ during a loading cycle, was set to 0.1. For each type of specimen, the fatigue tests were performed under five selected loading levels *r* (60–85%) to attain a S–N curve. The loading level is defined as the ratio of the maximum applied stress *σ*_max_ adopted in the fatigue test to the ultimate strength *σ*_uts_ obtained in the quasi-static test. The identical test was repeated twice to obtain the reliable fatigue data. The fatigue life *N_f_* is defined as the number of cycles corresponding to the specimen separation. The fatigue test was interrupted when the loading cycles exceeded 10^6^ cycles, and the specimen was considered to have infinite fatigue life. The temperature of the specimen was monitored using an infrared thermometer (8877AZ, AZ Instrument Corp., Taichung, Taiwan) during the fatigue test, and the maximum temperature rise of the specimens was found to be within 3 °C.

Since delamination is the main type of fatigue damage of the composite laminates, the mode I interlaminar fracture toughness *G*_IC_ was measured herein to evaluate the interlaminar property of the studied composite specimens with matrices modified under various nanofiller ratios. The tests were carried out based on the standard ASTM D5528 [43] by using the DCB specimens. The test was performed by controlling the crosshead speed at 5 mm/min, and the delamination length was visually monitored by using a microscope. The strain energy release rate *G*_I_ can be obtained using modified beam theory [43], which can be expressed by the following equation:

(1) $$G_{I}\;=\;\frac{3P\delta }{2w(a\;+\;\left|\Delta \right|)}$$

where *P* is the load, *δ* is the displacement of crosshead, *w* is the width of the specimen, *a* is the length of delamination, and ∆ is the correction factor of crack length, which can be obtained by fitting the relationship between the cubic root of specimen compliance *C*^1/3^ and the delamination length *a*, using the following equation:

(2) $$C^{1/3}\;=\;m(a\;+\;\left|\Delta \right|)$$

where *m* is a constant. The mode I fracture toughness can be obtained by substituting the critical load into Equation (1). Note that only the initial fracture toughness was measured in the study.

After the aforementioned tests, the fracture surfaces of the studied specimens were examined using a field emission scanning electron microscope (SEM) (S-4800, Hitachi Ltd., Tokyo, Japan) to determine the reinforcement mechanism of the employed nanoparticles on the studied mechanical properties.

## 3. Results

### 3.1. Quasi-Static Mechanical Properties

**Figure 4:**
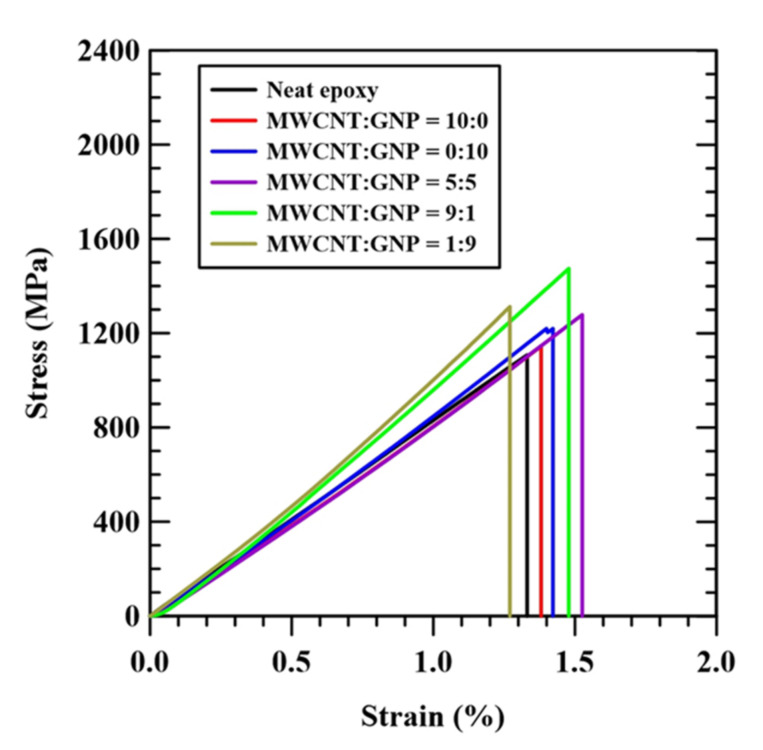
Examples of stress–strain curves for the nano-CFEP laminate specimens with matrices modified under various nanofiller ratios.

**Figure 5:**
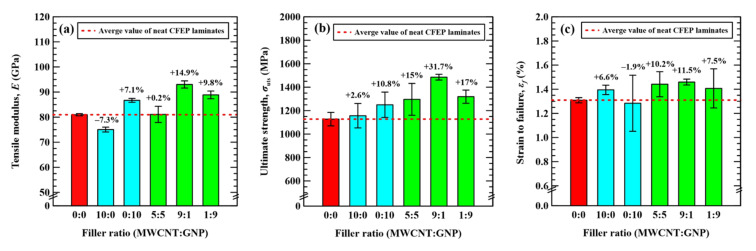
Effect of nanofiller ratio on the (**a**) tensile modulus, (**b**) ultimate strength, and (**c**) strain to failure of the studied nano-CFEP laminate specimens.

Figure 4 shows the representative stress–strain curves of all types of specimens with a matrix modification by MWCNTs and GNPs under various nanofiller ratios. For each type of specimen, the stress and strain obtained in the monotonically tensile tests followed a linear relationship, and the specimen fractured rapidly when the stress reached the peak value. Three mechanical properties, i.e., tensile modulus *E,* tensile strength *σ*_uts_, and strain at fracture *ε*_f_, are defined as the initial slope of the stress–strain curve, the stress at the peak point of the stress–strain curve, and the engineering strain corresponding to the fracture of specimen, respectively. The experimental data of these three properties obtained from the stress–strain curves are listed in Table 1. Figure 5 shows the comparison of these three mechanical properties of all types of specimens with matrix modification under different nanofiller ratios. As indicated in Figure 5a,b, the tensile modulus of the specimens modified by CNTs only decreases by 7.3%, and the tensile strength increases by 2.6%, compared to the reference specimens. Only the dispersal of CNTs in the matrix has reverse and slight effects on the tensile modulus and the strength of the neat CFEP specimen, respectively. However, the modification of a matrix by using GNPs only increases the tensile modulus and strength of the CFEP laminates by 7.1 and 10.8%, respectively.

The effect of embedding hybrid nanoparticles in the matrix on the modulus and strength depends on the adopted nanofiller ratios. The hybrid nano-CFEP laminate specimens with matrices modified under extreme MWCNT:GNP ratios, such as 1:9 or 9:1, present a significant synergistic effect on the tensile modulus and strength. The modulus and strength of the aforementioned hybrid nano-CFEP laminate specimens are higher than those with individual types of nanoparticles. the tensile modulus and strength of the specimens modified with hybrid nanoparticles under a MWCNT:GNP ratio of 9:1, especially, are the highest among the hybrid nano-CFEP laminate specimens modified under various nanofiller ratios. The modulus and strength increase by 14.9 and 31.7%, compared to the neat CFEP specimens, respectively. The tubular MWCNTs between the flake-like GNPs provides a bridging effect for load transfer, further improving the mechanical properties.

Furthermore, adding the nanoparticles under a MWCNT:GNP ratio of 5:5 in the matrix has no effect on the tensile modulus. Despite the fact that the improvement in tensile strength for dispersing hybrid nanoparticles under an even nanofiller ratio in the matrix is more significant than the employment of individual types of nanoparticles, the synergistic effect of hybrid nanoparticles under a MWCNT:GNP ratio of 5:5 on the tensile strength is lower than those under extreme nanofiller ratios.

As shown in Figure 5c, adding MWCNTs to the matrix is beneficial to the elongation of the CFEP laminate composites. The fracture ductility of the MWCNT-modified CFEP laminate specimens displays a 6.6% increase, compared to that of the neat CFEP laminate specimens. However, dispersing GNPs in the matrix presents a detrimental effect on the elongation of the specimens. The fracture ductility of the GNP-modified CFEP laminate specimens decreases by 1.9%, compared to that of the neat CFEP laminate specimens. The dispersion of hybrid nanoparticles displays a significant effect on the ductility of the CFEP composites. Figure 5c shows that the fracture ductility of the hybrid nano-CFEP laminate specimens are higher than that of the CFEP laminate specimens with individual types of nanofillers. Furthermore, the synergistic effect increases with the fraction of CNTs in the two nanofillers. That is, the hybrid nano-CFEP laminate specimen modified under a nanofiller ratio of 9:1 has the highest elongation, followed by that under a nanofiller ratio of 5:5, and the specimens modified under a nanofiller ratio of 1:9 have the lowest elongation.

### 3.2. Fatigue Properties

**Figure 6:**
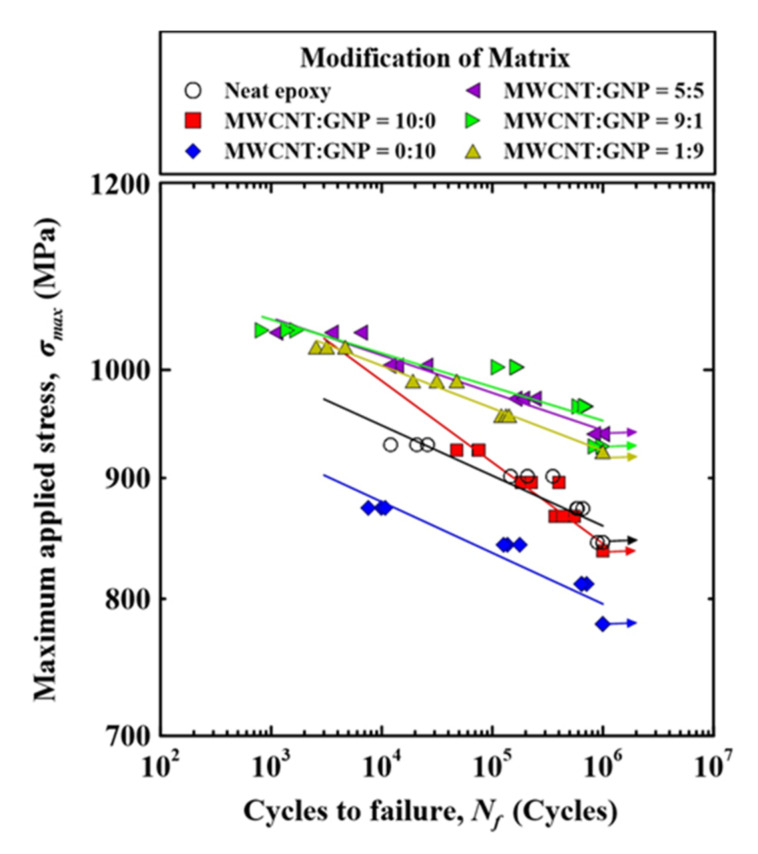
Relationships between the loading levels and the fatigue lives for the nano-CFEP laminate specimens with matrices modified under various nanofiller ratios.

Table 2 lists the experimental results of the studied nano-CFEP laminate specimens obtained in the fatigue tests. The relationships between the maximum applied stress *σ*_max_ and fatigue life *N_f_* of the specimens with matrices modified under various nanofiller ratios are plotted in Figure 6. For each type of specimen modified under different nanofiller ratios, a power-law model was employed to describe the relationship between the maximum applied stress and fatigue life data by curve fitting:

(3) $$\sigma_{\max}\;=\;aN_{f}^{b}$$

where *a* is the fatigue strength coefficient and *b* is the fatigue strength exponent. The material constants *a* and *b* depend on the applied nanofiller ratios of the studied specimens.

The fitting results were evaluated using the coefficients of determination *R*^2^. The values of *R*^2^ are also listed in Table 2. It was found that all the values of *R*^2^ for all types of specimens were higher than 0.8, demonstrating that the power-law model is appropriate to correlate the stress–life relationship of the studied CFEP laminate specimens. The fitting results of the power-law model are also plotted in Figure 6, which present as straight lines in the log–log scale diagram. Comparing the S–N curves of the specimens with matrices modified under various nanofiller ratios shows that adding individual types of nanoparticles to the matrix had no obvious effect on the improvement in fatigue strength of CFEP laminate specimens. Even the specimens with matrices modified by GNPs displayed a reversed effect on the improvement of the fatigue strength. The agglomeration of nanoparticles was considered as the reason for the poor performance in fatigue strength. On the other hand, the matrix modification using hybrid nanoparticles displayed a strong effect on the enhancement of the fatigue strengths of the CFEP laminate specimens. Furthermore, the slopes of the S–N curves for the specimens modified by hybrid nanoparticles were lower than those modified by individual types of nanoparticles, indicating that the fatigue strength of the hybrid nano-CFEP laminate specimens was more sensitive to the magnitudes of applied stress.

**Figure 7:**
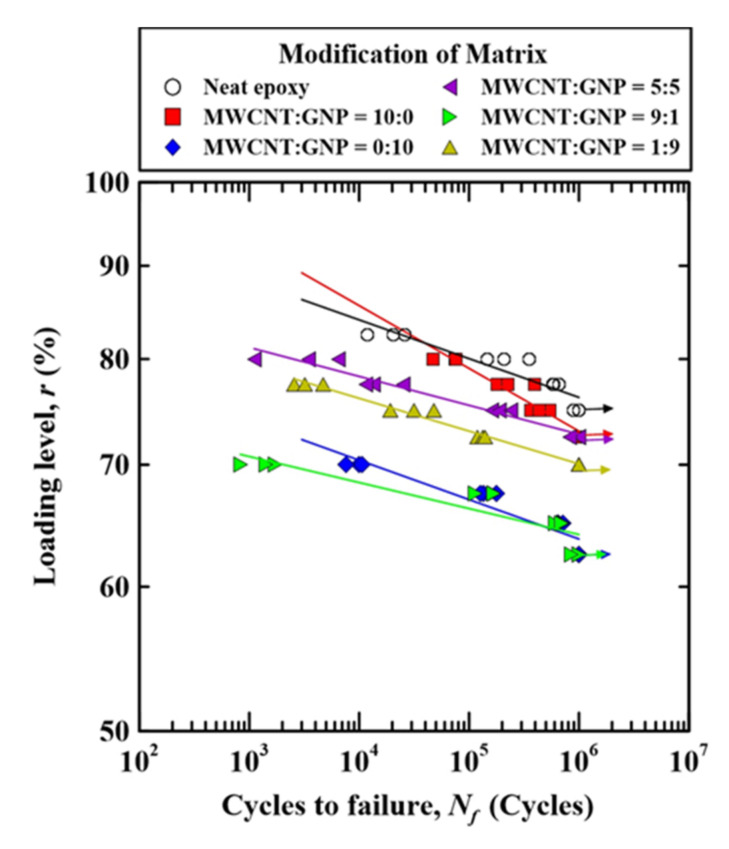
Relationship between the loading levels and the fatigue lives for the nano-CFEP laminate specimens with matrices modified under various nanofiller ratios.

Figure 7 displays the relationships between the loading levels and fatigue lives of the nano-CFEP laminate specimens with matrices modified under various nanofiller ratios. The figure is plotted by replacing the vertical axis of Figure 6 from the applied maximum stress to the loading level. It was found that dispersing nanoparticles in the matrix can reduce the loading level required to reach a certain fatigue life. The loading level for the nano-CFEP laminate specimen with matrix modified under a MWCNT:GNP ratio of 9:1 decreases by approximately 20%, compared to that for the neat CFEP laminate specimens.

**Figure 8:**
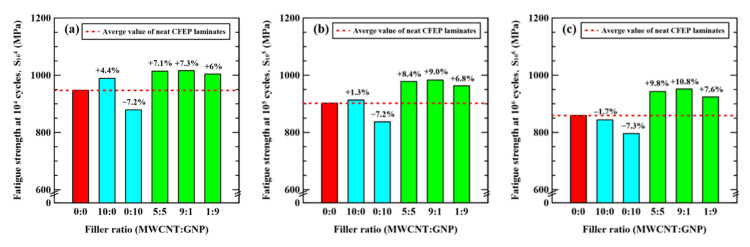
Fatigue strengths at (**a**) 10^4^, (**b**)10^5^, and (**c**) 10^6^ fatigue-life cycles for the nano-CFEP specimens modified under different nanofiller ratios.

To further analyze the effect of hybrid nanoparticles on the fatigue resistance at various fatigue-life cycles, the comparison of fatigue strengths corresponding to 10^4^, 10^5^, and 10^6^ fatigue-life cycles for all types of specimens with matrices modified under different nanofiller ratios was made in Figure 8. No matter what fatigue-life cycle is considered, adding only MWCNTs to the matrix had a slight influence on the fatigue strengths at various fatigue-life cycles. However, the nano-CFEP specimens modified with GNPs only presented lower fatigue strengths at different fatigue-life cycles than the neat CFEP laminates. Modifications of the matrix by hybrid nanoparticles showed a beneficial impact on the fatigue strengths. The improvements in fatigue strength for the laminate specimens modified under nanofiller ratios of 9:1 and 5:5 are almost identical, and slightly higher than those of 1:9. Moreover, for each type of hybrid nano-CFEP laminate specimen modified under a specific nanofiller ratio, the improvement in fatigue strength increases with the corresponding fatigue-life cycles.

**Figure 9:**
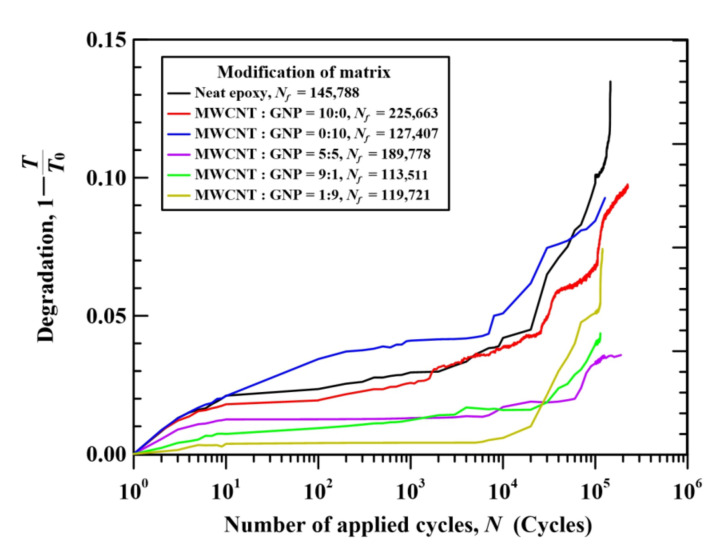
Variation of specimen degradation of the studied nano-CFEP laminate specimens with applied cycles.

To examine the effect of nanoparticles on the fatigue-damage evolution of CFEP laminates, the variations of specimen degradation with the applied fatigue cycles for the representative nano-CFEP laminate specimens modified under different nanofiller ratios were shown in Figure 9. For comparative purposes, the experimental data shown in Figure 9 were selected from the specimens with fatigue lives close to 200,000 cycles. The specimen degradation is defined based on the specimen stiffness and is expressed as 1−*T*/*T*_0_, where *T* and *T*_0_ are the stiffnesses corresponding to the *n*th cycle and the initial cycle in the fatigue test, respectively. The stiffness is obtained as the ratio of the stress range ∆*σ* to the strain range ∆*ε* in a fatigue cycle.

The degradation evolution of CFEP composites can be divided into three stages. At the first stage, the degradation increases rapidly due to the initiation and growth of matrix microcracks. The degradation becomes steadier and the increasing rate appears to be slower in the second stage. At this stage, the fiber/matrix debonding, delamination, and inner fiber fractures dominate the fracture mechanisms after the saturation of matrix microcracks. The degradation of CFEP laminate specimens increases rapidly again at the third stage until the final fracture. Figure 9 shows that the degradations of the CFEP laminate specimens with a neat epoxy matrix or with matrices modified with an individual type of nanofiller (MWCNT:GNP = 10:0 and 0:10) increase more rapidly than those modified with hybrid nanoparticles in the first stage. Furthermore, Figure 9 shows the degradations of the hybrid nano-CFEP laminate specimens shift to lower values, both at the first and second stages. This demonstrates that adding hybrid nanofillers to the matrix can reduce the rate of degradation of CFEP laminate specimens at the second stage, further improving the fatigue strength by retarding the evolution of the damage mechanism.

**Figure 10:**
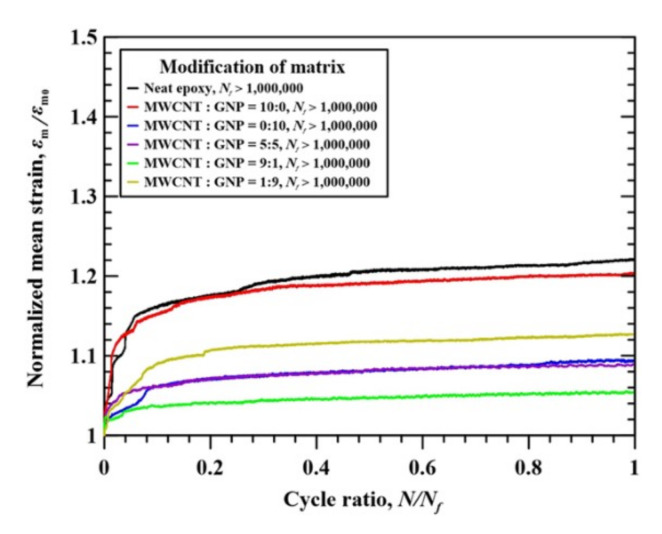
Variation normalized mean strain with the applied fatigue cycles for the studied CFEP laminate specimens modified under different nanofiller ratios.

Figure 10 shows the variation of normalized mean strain with the applied fatigue cycles for the studied CFEP laminate specimens with matrices modified under different nanofiller ratios. The normalized mean strain is obtained as the ratio of the mean strain at the *n*th cycle *ε*_m_ to the mean strain at the first cycle *ε*_m0_. The mean strain behaviors shown in this figure were obtained from the experimental data of specimens with fatigue lives larger than 10^6^ cycles. The mean strain behavior is an indicator of dynamic creep in the loading-controlled fatigue tests. The dynamic creep behavior becomes significant for the ductile materials when fatigue tested under asymmetric cyclic loading. Figure 10 shows that, for all types of CFEP laminate specimens with matrices modified under various nanofiller ratios, the normalized strains increase rapidly in the first 10% of life. After the transient behavior, the mean strain increases slightly with the applied cycle. The increase of the normalized mean strains at 10^6^ cycles is within 5%, compared to the steady values, implying that all types of the studied CFEP laminate specimens show brittle characteristics.

**Figure 11:**
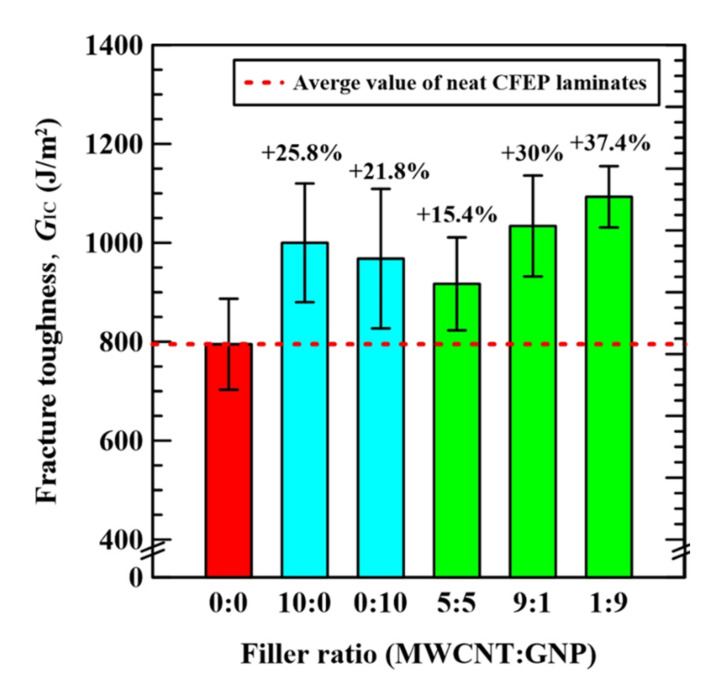
Variation of the mode I fracture toughness *G*_IC_ of the nano-CFEP specimens with the applied nanofiller ratios in the preparation of specimens.

The experimental results of mode I fracture toughness *G*_IC_ of the nano-CFEP laminate specimens with matrices modified under different nanofiller ratios are listed in Table 3. The variation of *G*_IC_ with the applied nanofiller ratios in the matrix modification of CFEP laminate specimens is plotted in Figure 11. It is evident that the values of fracture toughness for the CFEP laminate specimen with individual types of nanofillers are higher than the ones with a neat epoxy matrix. The matrix modifications using only MWCNTs and GNPs increase the fracture toughness of neat CFEP laminates by 25.8 and 12.8%, respectively. Moreover, the hybrid nano-CFEP laminate specimens with matrices modified under extreme nanofiller ratios (1:9 and 9:1) display more significant improvements in fracture toughness than those modified by individual types of nanofillers. The hybrid nano-CFEP laminate specimens with matrices modified under the MWCNT:GNP ratios of 9:1 and 1:9 present a 30 and 37.4% improvement in fracture toughness, respectively, compared to the neat CFEP laminate specimens. The synergistic effect of hybrid nanofillers with specific filler ratios on the interlaminar strength of CFEP laminates is verified by the fracture toughness tests, implying that the superior fatigue strength of hybrid nano-CFEP laminate specimens is expectable.

### 3.3. Observation of Fracture Surfaces

**Figure 12:**
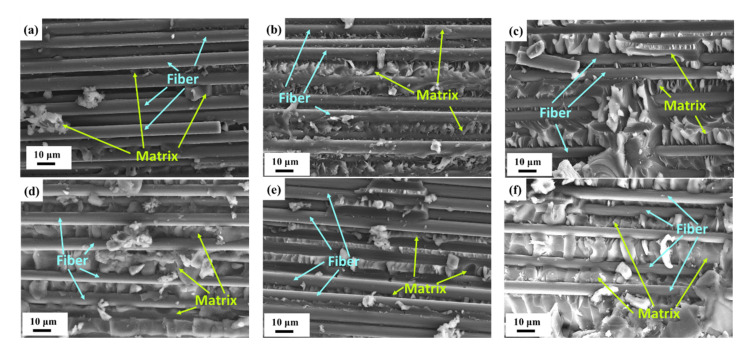
SEM images of fracture surfaces for the CFRP laminate specimens with matrices modified under the MWCNT:GNP ratios of (**a**) 0:0; (**b**) 10:0; (**c**) 0:10; (**d**) 5:5; (**e**) 9:1; and (**f**) 1:9.

Figure 12a–f show the representative SEM images of the fracture surfaces obtained after the fatigue tests for the CFEP laminate specimens with matrices modified under the MWCNT:GNP ratios of 0:0, 10:0, 0:10, 5:5, 9:1, and 1:9, respectively. The fracture surface of the neat CFRRP laminate specimen, shown in Figure 12a, displays a smooth appearance in the matrix, implying that no obstacles are encountered when the cracks propagate in the matrix. The rough fracture surface of the CNT-modified CFRP laminate specimen is observed (Figure 12b). The main reason for the formation of this uneven feature comes from the pullout of MWCNTs, and the deflection effect of the cracks provides secondary contributions. Figure 12c–f show the regular ripple-like features in the fracture surfaces of the matrix, indicating that the crack deflection effect is significant for these specimens with matrices modified by GNPs. The formation of this feature surface was due to the change of crack front direction or bifurcating when encountering the nanoparticles. This means that a higher energy consumption was needed to develop the characteristic fracture surface, further improving the fatigue strength [45,46,47,48]. Moreover, as shown in Figure 12b–f, lots of broken resin lumps adhered to the fracture surfaces of the specimens with matrices modified by the nanoparticles, implying that the bridging effect of the nanoparticles at the fiber/matrix interfaces improves the adhesion between the fiber and the matrix, further increasing the resistance to the interfacial debonding.

**Figure 13:**
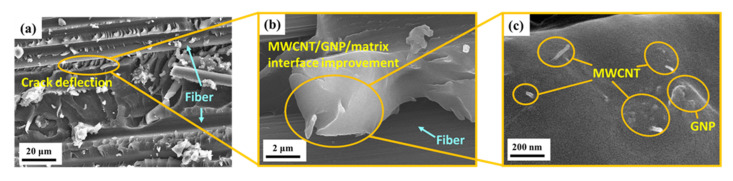
(**a**) Crack deflection observed from the fracture surface of the nano-CFEP laminate specimen modified under a MWCNT:GNP ratio of 9:1; (**b**) 3D cluster of MWCNTs and GNPs at the fiber/matrix interface; and (**c**) enlarged image of (**b**).

**Figure 14:**
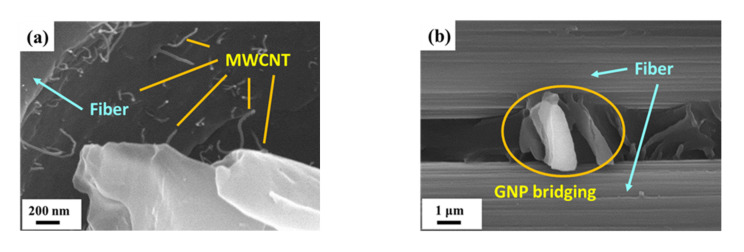
(**a**) MWCNT pullout and bridging observed from the matrix of the nano-CFEP laminate specimen modified under a MWCNT:GNP ratio of 9:1, and (**b**) GNP bridging observed from the fracture surface of the nano-CFEP laminate specimen modified under a MWCNT:GNP ratio of 0:10.

Figure 13 shows the local enlarger image observed at the site of the wavy fracture surface of another nano-CFEP specimen with a matrix modified under a MWCNT:GNP ratio of 9:1. Except for the crack deflection effect, the 3D cluster, consisting of MWCNTs and GNPs at the fiber/matrix interface, provided another enhancement mechanism to prevent the fiber–matrix debonding. Figure 14a,b present the SEM images of the fatigued nano-CFEP laminate specimens with matrices modified under the MWCNT:GNP ratios of 9:1 and 0:10, respectively. Figure 14a shows the pushout and bridging effects of MWCNTs in the matrix [40]. The bridging effect of GNPs at the interface between the polymer matrix and the fiber is shown in Figure 14b, which improves the interfacial strength and increases the resistance to debonding accordingly [4].

## 4. Discussion

**Figure 15:**
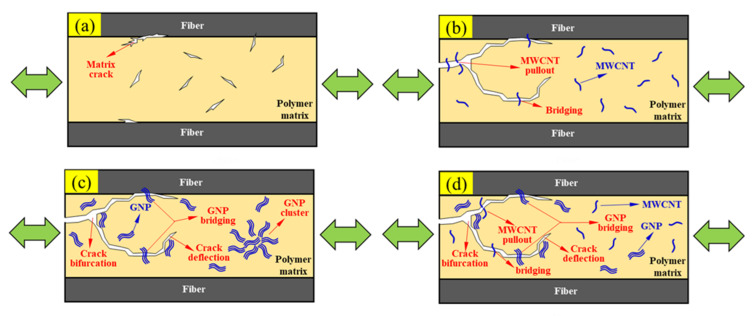
Schematic illustration of the fatigue improvement mechanisms for the (**a**) neat CFEP laminate specimen, (**b**) nano-CFEP laminate specimen modified with MWCNTs only, (**c**) nano-CFEP laminate specimen modified with GNPs only, and (**d**) nano-CFEP laminate specimen modified with hybrid nanoparticles.

The fatigue improvement mechanisms of the studied CFEP laminate specimens are schematically illustrated and explained in Figure 15. For the neat CFEP composites, the multiple microcracks initiate in the matrix at the beginning stage. These microcracks continue to grow and link to form the main crack under the cyclic loading. Once these main cracks propagate to the face/matrix interface, the interfacial debonding can be easily evolved to delamination and lead to the final fracture (Figure 15a). For the nano-CFEP modified with MWCNTs only, the bridging and pullout effects of tubular shaped nanoparticles provide resistance to the microcrack propagation at the beginning stage [49] and prevent the development of interfacial debonding at the following stage of fatigue damage evolution (Figure 15b). For the CFEP laminates modified by GNPs only, the crack deflection effect caused by the direction change or by the bifurcation of the crack front is the main enhancement mechanism at the microcrack development stage. Moreover, the flake shape of the GNPs can provide an excellent bridging effect to retard the propagation of interfacial cracks at the fiber/matrix debonding stage [4] (Figure 15c). It should be noted that the agglomeration of GNPs due to the strong interlayer π–π bond and Van der Waal force has a detrimental effect on the fatigue strength of the GNP-modified CFEP laminate specimens. The stress concentration resulting from the GNP clusters reduces the fatigue strength significantly [39,50]. For the CFEP specimens modified by the hybrid nanoparticles, the synergistic effect on the fatigue strength can be obtained by combining the characteristic fatigue-resistance mechanisms of individual nanoparticles together. Furthermore, adding the appropriate amount of MWCNTs to the GNP-rich polymer matrix can prevent the agglomeration of GNPs because the MWCNTs can disperse the GNPs effectively and provide advantageous load-transfer networks between the GNPs to eliminate the stress concentration effect [39,50] (Figure 15d).

It is well known that the agglomeration of nanoparticles decreases the mechanical properties of nanocomposites due to the stress concentration effect. Despite that the 2D structures of GNPs provide large contact areas with the polymer matrix, the anti-fatigue performance of GNP-modified CFRP laminates is lower than the CNT-modified ones (Figure 8). At the stage of the formation of microcracks, the stress concentration resulting from the agglomeration of GNPs accelerates the initiation of cracks, further decreasing the fatigue strength of the CFRP laminates. The chemical or mechanical dispersion techniques (other than ultrasonic homogenization and planetary centrifugal mixing) employed in this study should be considered in the future works to solve the agglomeration problem. Oppositely, the clusters of nanoparticles may be the effective obstacles to suppress the crack propagation. As mentioned above, the crack deflection effect of nanoparticles is the main reinforcement mechanism for fatigue strengths, especially at the stage of delamination growth. Hence, the study of the delamination growth rate of CFRP should be performed in the future to clarify the different roles played by the nanoparticle agglomeration at the initiation and propagation stages.

It is also noted that replacing few nanoparticles of the CFRP laminate specimens modified by individual types of nanoparticles with the second-phase nanoparticles can improve the mechanical properties remarkably. Comparing the monotonic and fatigue experimental results, between the specimens modified under the nanofiller ratios of 10:0 and 9:1, has verified this inference. Similar comparison results can also be observed between the specimens modified under the nanofiller ratios of 0:10 and 1:9. Adding 2D nanoparticles to the nanocomposites modified by 1D nanoparticles can increase the contact area with the matrix, further improving the mechanical properties. On the other hand, adding a small amount of 1D nanoparticles to the matrix of 2D nanoparticle-modified CFRP laminates can separate the 2D particles and improve the dispersion of the 2D nanoparticles. Moreover, the characteristic 1D structure of CNTs between GNPs constitutes an excellent network for load transfer. The experimental data of the CFRP specimens modified under the filler ratio of 5:5 reveals that the excessive addition of second-phase nanoparticles has a reversed influence on the synergistic performance of hybrid nanoparticles on the mechanical properties. Although the experimental data support that the hybrid nano-CFRP laminates with modifications under the nanofiller ratios of high discrepancy (1:9 or 9:1) display excellent mechanical properties in simulation; more theoretical or simulative works need to be performed to quantify the effect of nanofiller ratios of hybrid nanoparticles on the studied mechanical properties in the future.

## 5. Conclusions

The effect of adding MWCNTs and GNPs to the matrix on the tensile quasi-static and fatigue properties of CFEP laminate specimens has been experimentally investigated herein. Some conclusions can be summarized as follows:

1. The nano-CFEP laminate specimens with matrices modified under a MWCNT:GNP ratio of 9:1 have a higher monotonic modulus and strength than the CFEP laminate specimens with matrices modified under other conditions;
2. Adding individual types of nanoparticles has a slight influence on the tensile fatigue strength of CFEP laminates. However, the CFEP laminate specimens with matrices modified by hybrid nanoparticles display a significant improvement in fatigue strength compared to the neat CFEP specimens or specimens modified with individual types of nanoparticles;
3. Examining the evolution of stiffness-based degradation shows that polymer matrices modified by hybrid nanoparticles can effectively shift the degradation to lower values;
4. The synergistic effect of MWCNTs and GNPs on the mode I fracture toughness of CFEP composites has been experimentally verified, implying that the hybrid nanoparticle system employed in the matrix modification can improve the resistance to interfacial debonding and delamination propagation;
5. The pushout effect of MWCNTs and the crack deflection effect of GNPs are the main enhancement mechanisms at the matrix microcrack dominant stage. The bridging effect of nanoparticles at the fiber/matrix interfaces retards the growth of interfacial debonding, further improving the fatigue strength.

This study confirmed that adding MWCNT and GNP hybrids under appropriate nanofiller ratios can improve the fatigue strength of bulk CFRP laminates remarkably. The synergistic effect of hybrid nanoparticles on the delamination propagation rate of cracked CFRP laminate specimens deserves to be investigated in depth in the future. Furthermore, the synergistic effect of other hybrid nanofillers with different nano-dimensionalities on the static and fatigue properties of CFRP composites is another topic worth studying. Since the agglomerations of nanoparticles are detrimental to the mechanical properties, the innovative chemical or mechanical techniques for the dispersion of nanoparticles should be developed continuously to obtain the uniform dispersion of nanoparticles in the polymer matrix. The corresponding reinforcement mechanisms of the employed hybrid nanoparticles on the fatigue behavior also need further observation and discussion to verify the contribution of each type of nanoparticle.

## Figures and Tables

**Table 1:** Quasi-static mechanical properties of the studied nano-CFEP laminate specimens with matrices modified under various nanofiller ratios.

Nanofiller Ratio MWCNT:GNP	Mechanical Properties		
	Tensile Modulus *E* (GPa)	Tensile Strength *σ_uts_* (MPa)	Strain to Failure *ε_f_* (%)
Neat epoxy	80.96 ± 0.43	1128 ± 58	1.31 ± 0.02
10:0	75.05 ± 0.97	1157 ± 104	1.39 ± 0.04
0:10	86.67 ± 0.77	1250 ± 108	1.28 ± 0.23
5:5	81.09 ± 3.22	1296 ± 136	1.44 ± 0.10
9:1	93.01 ± 1.43	1486 ± 25	1.46 ± 0.02
1:9	88.86 ± 1.55	1319 ± 56	1.41 ± 0.16

**Table 2:** Experimental results of the studied nano-CFEP laminate specimens obtained in the fatigue tests.

Nanofiller Ratio MWCNT:GNP	Loading Level *r* (%)	Fatigue Life *N_f_* (cycles)	Fatigue Strength Coefficient *a*	Fatigue Strength Exponent *b*	Coefficient of Determination *R^2^*
Neat epoxy	75	890,023, 1,000,000, 1,000,000	1152	0.021	0.86
	77.5	585,644, 656,983, 579,416			
	80	145,788, 207,800, 353,534			
	82.5	26,145, 11,980, 20,593			
10:0	72.5	1,000,000, 1,000,000, 1,000,000	1358	−0.034	0.92
	75	547,691, 367,158, 431,649			
	77.5	398,070, 181,435, 225,663			
	80	74,476, 47,396, 75,509			
0:10	62.5	1,000,000, 1,000,000, 1,000,000	1073	−0.021	0.88
	65	649,877, 716,640, 64,2066			
	67.5	136,197, 127,407, 177,407			
	70	9939, 10,689, 7606			
5:5	72.5	1,000,000, 1,000,000, 819,407	1174	−0.016	0.96
	75	160,943, 189,778, 241,175			
	77.5	13,605, 11,602, 25,091			
	80	1106, 6440, 3487			
9:1	62.5	1,000,000, 1,000,000, 849,793	1159	−0.014	0.80
	65	609,748, 714,277, 686,646			
	67.5	168,684, 113,511, 167,219			
	70	829, 1718, 1415			
1:9	70	1,000,000, 1,000,000, 1,000,000	1185	−0.018	0.98
	72.5	119,721, 133,696, 140,635			
	75	31,506, 19,159, 47,737			
	77.5	2561, 3188, 4670			

**Table 3:** Experimental results of mode I fracture toughness of studied nano-CFEP laminate specimens with matrices modified under various nanofiller ratios.

**Nanofiller Ratio** **MWCNT:GNP**	Neat Epoxy	10:0	0:10	5:5	9:1	1:9
**Mode I fracture toughness** ***G***_**IC**_ **(J/m** ^**2**^ **)**	795 ± 92	1000 ± 120	968 ± 141	917 ± 94	1034 ± 102	1093 ± 62
